# Are CSF CXCL13 concentrations solely dependent on intrathecal production? A commentary on “Chemokine CXCL13 in serum, CSF, and blood–CSF barrier function”

**DOI:** 10.1186/s12987-021-00244-5

**Published:** 2021-02-25

**Authors:** Krista D. DiSano, Francesca Gilli, Andrew R. Pachner

**Affiliations:** Department of Neurology, Geisel School of Medicine & Dartmouth-Hitchcock Medical Center, One Medical Center Drive, Lebanon, NH 03756 USA

**Keywords:** Cerebrospinal fluid, Multiple sclerosis, CXCL13, Biomarkers

## Abstract

Pilz et al. (Fluids Barriers CNS 17:7; 2020) investigated how CSF CXCL13 concentrations are influenced by CXCL13 serum concentrations and blood-CSF barrier (BCSFB) function, comparing the impact of serum CXCL13 levels and Q_albumin_ (CSF albumin/serum albumin) on CSF CXCL13 among patients with CNS inflammation categorized as CXCL13 negative, low, medium, or high. Among all CXCL13 groups, their results showed no correlation between CSF CXCL13 concentrations and serum CXCL13 or Q_albumin_. The authors argue that, in contrast to other proteins, CXCL13 passage across the BCSFB does not occur, regardless of BCSFB function, and is instead solely influenced by intrathecal production. In contrast to the authors’ findings, in our studies including both non-inflammatory neurological disorders (NIND; n = 62) and multiple sclerosis (MS) patients we observed a significant correlation between serum CXCL13 concentrations and CSF CXCL13 concentrations. We review several observations which may underlie these contrasting results, including (1) the impact of serum CXCL13 concentrations on CSF CXCL13 in patients with lower intrathecal CXCL13 production and thus lower CXCL13 concentrations (i.e. NIND and MS), (2) the proposed diffusion dynamics of the small molecule CXCL13 across the BCSFB, and (3) differing definitions of negative versus elevated CSF CXCL13 concentrations determined by an assay’s relative sensitivity. In conclusion, we argue that for patients with moderately elevated CSF CXCL13 concentrations, serum CXCL13 concentrations influence CSF CXCL13 levels, and thus the appropriate corrections including incorporation of CSF/serum ratios and Q_albumin_ values should be utilized.

## Background

There has been increased attention to candidate biomarkers in the cerebrospinal fluid (CSF) in neurological diseases. For biomarkers derived from the periphery or CNS, including inflammatory proteins, the relationship between serum and CSF levels of these proteins are an important concept. CXCL13, a 10 kD chemokine, is a candidate CSF biomarker in MS [1]. CXCL13 is a conventional lymphoid tissue chemokine and elevated serum CXCL13 is associated with germinal center formation [2, 3]. CXCL13 is also ectopically produced in the central nervous system during inflammatory conditions [4, 5]. A recent manuscript published by Pilz et al. [6] concluded CXCL13 does not cross from the blood into CSF and is instead exclusively derived from intrathecal production. These results challenge the use of CSF/serum quotients or index calculations (i.e. (CSF analyte/serum analyte)/(CSF albumin/serum albumin), which are conventionally used to distinguish between passive transfer of proteins from the serum across the blood-CSF-barrier (BCSFB) into the CSF and intrathecal production.

## Main text

Pilz et al.’s conclusion that the BCSFB is completely closed to a small protein like CXCL13 runs counter to long experience of neuroscientists with the equilibrium between proteins in the blood and CSF [7], and would represent, if true, a remarkable and surprising exception. Serum is the source of 80% of all CSF proteins [8]. As noted by Reiber [9], “protein transfer from brain into CSF and from blood into CSF follows the laws of diffusion as a function of molecular size”. For CXCL13 to violate these laws is highly doubtful, especially considering that CXCL13 is a small molecule, i.e., a 10.3 kDa protein of 87 amino acids.

Second, the conclusions in the Pilz manuscript were not sufficiently supported by the data presented, especially for inflammatory conditions, such as multiple sclerosis (MS), where there is minimal, if any, BCSFB dysfunction, and there are small elevations of intrathecally produced CSF CXCL13 relative to the dramatic elevations seen in diseases such as Lyme neuroborreliosis (LNB) [10–12]. Thus, given that the Q_CXCL13_, i.e. the ratio of CSF to serum CXCL13, in “normals” and non-inflammatory controls, is approximately 0.025–0.1 [13], one would expect that serum levels of 64 to 82 pg/ml would be associated with CSF levels of approximately 2–8 pg/ml, if there is no intrathecal CXCL13 production. Only 9 of Pilz’s patients had serum levels higher than 250 pg/ml, and thus the serum contribution to the CSF level in 60/69 (87%) of the patients would be expected to be 25 pg/ml or less, a range that the authors considered “negative”. Thus, for most of the patients in the study who had neuroinflammatory conditions associated with high levels of intrathecal CXCL13 production, the contribution of serum diffusion across the BCSFB would be low relative to that produced intrathecally.

In MS, there is intrathecal production of CXCL13, but at a lower level. Thus, the diffusion of serum CXCL13 into the CSF contributes a larger fraction of CSF CXCL13 than in LNB or the other highly inflammatory conditions, such as meningitis and encephalitis, in the Pilz study. Thus, utilizing the CXCL13 index, which accounts for both serum concentrations and BCSFB function, provides better information than CSF CXCL13 concentrations alone [13].

Third, the prediction would be that, based on its molecular weight of 10.3 kDa, the Q_CXCL13_ for CXCL13 would be approximately 0.025–0.1, and this number has been confirmed in a number of studies [14, 15]. The Pilz manuscript authors state that they were “assuming diffusion dynamics across the BCSFB similar to albumin or immunoglobulin”, but those two molecules have much lower expected Q (CSF/serum) values since they are 7 to 15 times bigger than CXCL13, and thus the contribution of serum levels to CSF levels for these two large molecules is much less.

Fourth, Pilz et. al. were somewhat limited in analysis of situations with relatively low CSF CXCL13 by their use of the Euroimmun CXCL13 ELISA as their method of measurement. This ELISA, which is widely used for LNB [13, 16, 17], is not very sensitive, with a lower limit of quantitation (LLOQ) of 10.7 pg/ml [18]. Our testing of the Euroimmun ELISA has confirmed this high LLOQ, and 36% of CSFs from randomly chosen MS patients were below the LLOQ, and could not be accurately quantitated.

**Fig. 1:**
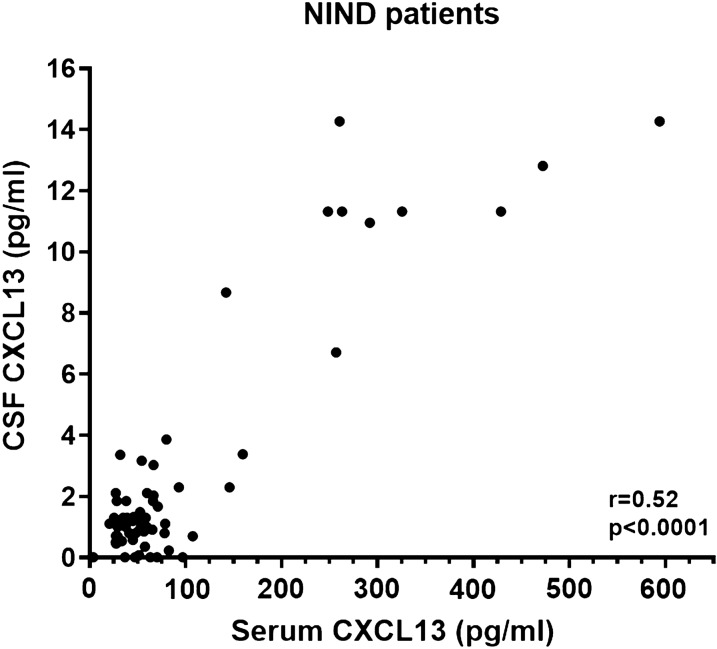
Serum CXCL13 correlates with CSF CXCL13 levels in NIND patients. CSF and serum CXCL13 (pg/ml) were measured utilizing a human chemokine CXCL13 bead-based immunoassay (Bio-plex Pro™; Bio-Rad, Hercules CA; LLOQ 0.7 pg/ml) in non-inflammatory neurological disease controls (NIND; n = 62). Datasets were tested for normality and were determined to be non-parametric. Correlations were performed comparing serum and CSF CXCL13 values utilizing a Spearman’s rank order correlation for non-parametric data to determine the spearman’s rank correlation coefficient (r) and statistical significance. All statistical analyses were performed using GraphPad Prism version 7.00 (GraphPad, San Diego, CA). P values < 0.05 were deemed to be statistically significant

Finally, there is strong evidence from our laboratory in non-inflammatory neurological conditions (NIND) that blood levels of CXCL13 contribute to CSF concentrations. Unlike the chemokines CXCL10, CCL2, CCL19, and CXCL12, among others, which are constitutively produced in the CNS [14, 19–21], CXCL13 is not produced intrathecally in non-inflammatory neurological conditions. Thus, analyzing the correlation of CSF to serum CXCL13 will give an accurate picture of the contribution of serum diffusion across the BCSFB into CSF. When we analyzed this recently using a CXCL13 bead-based immunoassay (Bio-plex Pro™; Bio-Rad, Hercules CA; LLOQ 0.7 pg/ml) as previously described [13], we found a strong correlation of CSF to serum CXCL13 concentrations (Fig. 1; r = 0.52 (CI = 0.29–0.68); p < 0.0001) in NIND patients (n = 62). NIND patients included were headache syndromes (n = 24), non-inflammatory neuropathies (n = 16), cognitive dysfunction (n = 5), epilepsy (n = 4), and other non-inflammatory neurological illnesses (n = 13) including Arnold-Chiari deformity (n = 1), dizziness (n = 1), Horner syndrome (n = 1), facial numbness (n = 2), fasciculation (n = 1), leg weakness (n = 3), movement disorders (n = 2), post-concussive syndrome (n = 1), and ischemic stroke (n = 1).

## Conclusion

In conditions in which intrathecal production of CXCL13 is exuberant, such as in LNB and other highly neuroinflammatory conditions, the contribution of serum CXCL13 to CSF CXCL13 concentrations is relatively slight, and is dwarfed by intrathecal production, but in many other conditions such as in MS and in non-inflammatory neurological diseases [17], diffusion from the serum into the CSF across the BCSFB must be factored in.

## Data Availability

Data is available upon request to the corresponding author.

## Abbreviations

BCSFB: Blood-cerebrospinal fluid-barrier
CI: Confidence interval
CNS: Central nervous system
CSF: Cerebrospinal fluid
ELISA: Enzyme-linked immunoassay
kDa: Kilodalton
LLOQ: Lower limit of quantitation
LNB: Lyme neuroborreliosis
MS: Multiple sclerosis
NIND: Non-inflammatory neurological disease
